# Preparation of stable enteric folic acid-loaded microfiber using the electrospinning method 

**DOI:** 10.22038/IJBMS.2022.61563.13625

**Published:** 2022-03

**Authors:** Abbas Akhgari, Pariya Iraji, Niloufar Rahiman, Akram Hasanzade Farouji, Mohammadreza Abbaspour

**Affiliations:** 1 Department of Pharmaceutics, School of Pharmacy, Mashhad University of Medical Sciences, Mashhad, Iran; 2 Targeted Drug Delivery Research Center, Pharmaceutical Technology Institute, Mashhad University of Medical Sciences, Mashhad, Iran; 3 Nanotechnology Research Center, Pharmaceutical Technology Institute, Mashhad University of Medical Sciences, Mashhad, Iran; 4 Department of Pharmaceutical Nanotechnology, School of Pharmacy, Mashhad University of Medical Sciences, Mashhad, Iran

**Keywords:** Electrospinning, Enteric, Eudragit, Folic acid, Microfiber

## Abstract

**Objective(s):**

Folic acid is an essential vitamin, labile to hydrolysis in the acidic environment of the stomach with low water solubility and bioavailability. In order to solve these problems, enteric oral folic acid-loaded microfibers with a pH-sensitive polymer by electrospinning method were prepared.

**Materials and Methods:**

Electrospinning was performed at different folic acid ratios and voltages. Fibers were evaluated in terms of mechanical strength, acidic resistance, and drug release. Additionally, DSC (Differential Scanning Calorimetry), FTIR (Fourier-transform infrared spectroscopy), and XRD (X-ray diffraction) analyses were performed on the optimal formulation.

**Results:**

Drug ratio and voltage had a considerable effect on fibers’ entrapment efficiency, acid resistance, and mechanical strength. Based on the obtained results, the optimum formulation containing 1.25% of the drug/polymer was prepared at 18 kV. The entrapment efficiency of the optimal sample was above 90% with an acid resistance of higher than 70%. The tensile test confirmed the high mechanical properties of the optimum microfiber. DSC and XRD tests indicated that folic acid was converted to an amorphous form in the fiber structure and the FTIR test confirmed the formation of a chemical bond between the drug and the polymer. The release of the drug from the optimal fiber was about 90% in 60 min.

**Conclusion:**

In conclusion, the optimal formulation of folic acid with proper mechanical properties can be used as a candidate dosage form for further bioavailability investigations.

## Introduction

Oral drug delivery is the most desirable drug administration route due to its higher patient compliance (1). In contrast, this kind of drug administration makes the drug susceptible to the acidic and enzymatic environment of the gastrointestinal tract (2). The drugs labile to the acidic environment of the stomach should reach the duodenum to be absorbed. For this purpose, acid-resistant drug delivery systems (enteric-coated drugs) are beneficial. This enteric coating protects the labile drug from degradation and hydrolysis by the gastric acid (3). 

The pH-responsive drug delivery systems are designed to bypass the acidic environment of the gastrointestinal tract. Eudragit^®^ S 100 is a pH-sensitive polymer, used for enteric coating or as a vehicle for drug delivery systems. The polymer has some unique properties like plasticity and significant pH sensitivity. It is an anionic copolymer (the mixture of methacrylic acid and methacrylate) with high conductivity (4, 5). The free carboxylic acid groups in that polymer make it pH-sensitive, insoluble in acidic environments and water, and soluble in the intestinal environment with pH above 7 (6). In the gastric acidic environment, this polymer acts as a polyelectrolyte and preserves its condensation and hydrophobicity due to protonation of carboxylic acid groups. Exposure to the higher pH leads to ionization of the carboxyl groups and hydrolyzation of the hydrogen bond, which makes the polymer inflated and causes it to release its content throughout the ileum and colon (7, 8)following the Langmuir model in a typical adsorption at solid/liquid interface (pseudo-second-order model. This enteric polymer has been widely used for the production of electrospun formulations of different drugs such as aceclofenac (9), methylprednisolone (10), and aspirin (11).

Folic acid, as a member of the vitamin B group and a vital vitamin in the prevention of megaloblastic anemia and neural tube defects in pregnant women (12), is rapidly absorbed from the gastrointestinal tract. The duodenum and jejunum are the main areas for folic acid absorption (13). The pure form of this vitamin is poorly soluble (~2 mg/ml) (14, 15), sensitive to alterations in pH and temperature, and also exposure to oxygen and light. Due to these characteristics, the development of proper drug delivery systems is a major challenge for protecting the drug from an acidic environment and increasing its solubility (16). Encapsulation of folic acid in micro/nanofibers could be a promising strategy for addressing the mentioned hurdles (17). 

Nanofibers are fiber-like structures with diameters in the nanoscale range (18). In addition to their nanosized diameter, high surface-to-volume ratio, high porosity, small pore size, high mechanical strength, and functional diversities are their other advantages (19). Various controlled drug release profiles, such as sustained, immediate, and biphasic releases, could be also attained by electrospun nanofibers (20). Moreover, nanofiber fabrication exploiting electrospinning is a straightforward process (21). In comparison to other spinning techniques, electrospinning is a cost-effective method for producing ultra-fine nanofibers with a diameter within the range of nanometer, controlled surface morphology, and high loading capacity for different drugs (18). 

The polymer solution concentration, conductivity, viscosity, and surface tension, along with the applied electric potential, collector distance, feed rate, temperature, and the relative humidity in the spinning chamber, are the determinative parameters in the morphology of the obtained nanofibers. The polymer solution exposure to a high voltage results in charged polymeric droplets (22, 23). Subsequently, the droplets are extruded through a nozzle in the electrospinning apparatus and are elongated by the electrostatic force effect (24, 25). The nanofibers are formed after solvent evaporation from the droplets and then collected on the collector plate (26, 27).

In this study, our main objective is to prepare folic acid-loaded micro/nanofibers by the electrospinning method using Eudragit^®^ S 100 as an enteric nanosystem for enhancement of folic acid stability and bioavailability. According to the susceptibility of folic acid to acidic conditions and its low aqueous solubility, designing a protective formulation to release folic acid in the small intestine and its solubility increment helps to improve absorption of the drug. Besides, by assessing various critical parameters, such as feed rate, applied voltage, and quantity of the polymer and the drug, the effects of these parameters on different properties of the resulting fibers could be determined.

## Materials and Methods

***Materials***

Folic acid (Sigma, USA), Eudragit^®^ S 100 (ES) (Evonik, Germany), KH_2_PO_4_ (Merck, Germany), NaOH (Merck, Germany), HCl 37% (Merck, Germany), Ethanol 96% (Kimia Alcohol Zanjan, Iran) were obtained from the indicated sources.

***Experimental design***

The box-Behnken design was used for determination of experimental formulations, correlation between variables and responses, and selection of the optimum formulation. The independent variables studied in this research were the distance between the needle and collector, feed rate, and voltage. According to the previous observations, the folic acid concentration and voltage were considered as the influential variables in nanofibers’ characteristics (28), and their maximum and minimum levels were determined (Table 1). Considering variables and their levels, the response surface methodology was applied to obtain the study design using the Design-Expert 10 software package. According to the box-Behnken design with nine axial points and 0 center points, nine formulations were selected (Table 2).

***Preparation of spinning solutions***

The solution of Eudragit^®^ S 100 (10% w/v) was prepared in ethanol 96^°^ as the appropriate solvent. The polymer was dissolved in the solvent while stirring on a magnetic stirrer for 24 hr. Three different folic acid concentrations were prepared by addition of 5, 12.5, and 20 mg folic acid in 1.8 ml PBS (pH=6.8). Only 5 mg folic acid was completely dissolved in PBS (pH=6.8) but 12.5 and 20 mg folic acid could not dissolve completely and produce a uniform dispersion. The folic acid concentrations were then dispersed in the ethanolic solution of Eudragit^®^ S 100 to make the specified drug/polymer ratio (Table 2). After 24 hr of incubation at 4 ^°^C, the dispersions were reached to the desired volume with ethanol 96^°^ and were homogenized on a magnetic stirrer for another 1 hr. 

***Electrospinning process***

Electrospinning solutions/suspensions were loaded in 5 ml syringes. The feeding rate was controlled by a syringe pump (Uniaxial electrospinning machine, ES1000 model, Fanavaran Nano Meghyas Co, Iran) and fixed at 1.0 ml/h and the collector rotational speed was set at 200 rpm. A high voltage of 12-18 kV was applied, and a piece of aluminum foil was used on the collector to collect the ultrafine fibers with a horizontal distance of 150 mm from the needle tip. The electrospinning process was performed at room temperature (25.0±0.2 ^°^C) with 45% relative humidity. Electrospun microfibers were collected and stored at 4-8 ^°^C for further studies.

***Fibers’ evaluations***

*Scanning Electron Microscopy (SEM)*

The morphology of electrospun fibers manifested using a FESEM (MIRA3, TESCAN CO, Czech Republic) after sputter coating with the mixture of gold and palladium under vacuum. The electrospun fibers’ mean diameter was determined using Image J software on SEM micrographs. Besides, the images were used to investigate the morphology and assessment of their uniformity or bead formation.

*Elasticity and tensile strength tests*

Fibers with a dimension of 1 cm ×4 cm and a specified thickness were cut and placed between the two clips of the Hounsfield H50SK material testing machine equipped with a 1KN load cell, using a cardboard mold. The tensile test was performed at the speed of 5 mm/min and Young’s modulus, yield stress, and elongation were determined as well. This test was carried out in triplicate for each sample.

*Entrapment efficiency*

A specific amount of the microfibers was dissolved in phosphate buffer and their UV absorption was recorded in 281 nm (29) by a spectrophotometer (Shimadzu UV-1204, Shimadzu, Japan). The absorbance was converted to the drug’s concentration through the standard equation and compared with the theoretical content of the drug in each microfiber (equation (1)). This test was performed in triplicate for each sample.

Entrapment efficiency (%)=Actual folic acid content in microfibers/Theoretical folic acid content in microfibers×100 (1)

*Acid resistance test*

A fiber sample with a specific weight was incubated in HCl (pH=1.2) at 37 ^°^C for 2 hr to mimic the gastric environment. The fibers were then dissolved in phosphate buffer, and their absorption was determined at 281 nm. Subsequently, the amount of remaining drug was calculated on the basis of the standard equation. This test was performed in triplicate for each sample.

*Selection of the optimum formulation*

The results of the performed assays on the samples were presented through the mathematical model using the Design-Expert software package. It demonstrated the correlation between variable factors (e.g., drug percentage and voltage) with fibers’ characteristics such as mean fiber diameter, strength and elasticity of fibers, entrapment efficiency, and acid resistance. 

After determination of the relationship between the factors and the desired responses, the optimum formulation was selected in terms of the minimum average diameter, maximum resistance to an acidic environment, and medium elasticity and strength. Subsequently, the following complimentary assessments were performed on the optimum selected microfiber (F8, according to Table 2).

*Drug release*

A piece of fiber equivalent to 375 µg of folic acid was incubated at 37 ^°^C in 30 ml phosphate buffer (pH=6.8) and the samples were collected at 5, 10, 15, 20, 30, 45, and 60 min time points. During the sampling procedure, the sampling volume was replaced with an equal volume of the fresh medium buffer, for maintaining the sink condition during the release study (30). The samples’ absorption was recorded by a spectrophotometer (Shimadzu UV-1204, Shimadzu, Japan) at 281 nm. The absorptions were converted to concentration with the standard equation, and the release percentage of the drug was calculated at different time points. The release analysis was performed in triplicate for each sample. 

*Differential scanning calorimetry (DSC)*

The DSC thermogram of the folic acid powder, Eudragit^®^ S 100, the physical mixture of drug and Eudragit^®^ S 100 (folic acid: polymer=1.25%), and a selected piece of microfiber were obtained. Three milligrams of each sample were placed in a specific aluminum pan and tested by the DSC device (model 82, Mettler Toledo, Switzerland). The target samples were heated at 35-350 ^°^C with a 10 ^°^C/min heating rate, and their thermal behavior was recorded versus the vacant aluminum pan as a reference.

*Fourier transform infrared spectroscopy (FTIR)*

To study the functional groups, the possible physical interferences, and chemical bonds between the drug and polymer, FTIR analysis was carried out. Three milligrams of folic acid powder, polymer powder, and the physical mixture of drug and Eudragit^®^ S 100 were tested by an FTIR spectrophotometer (Spectrum Two model, Perkin Elmer, USA) separately with a scanning range of 400 to 4000 cm^-1^ (31). The resulting data were recorded by PerkinElmer Spectrum software version 10.03.02.

*X-Ray diffraction (* *XRD)*

The XRD analysis was carried out for folic acid powder, Eudragit^®^ S 100 powder, the physical mixture of drug and Eudragit^®^ S 100, and the selected piece of microfiber. In the XRD apparatus (PW3710 model, Philips Analytical, the Netherlands), copper was utilized as an anode for radiation. The procedure was carried out in the θ interval of 4-60 degrees, 40 kV, and 30 mA (31). 

***Statistical analysis of data***

Linear regression and analysis of variance were used for investigation of the effects of the variable factors on each experimental response and their correlation. Design-Expert software was utilized for this purpose, and polynomial mathematical models were obtained. The final coefficients in this model were statistically significant. According to these mathematical models, three-dimensional diagrams were plotted for demonstrating the effect of variable factors on response.

## Results

***Fibers’ morphology***

**Figure 1:**
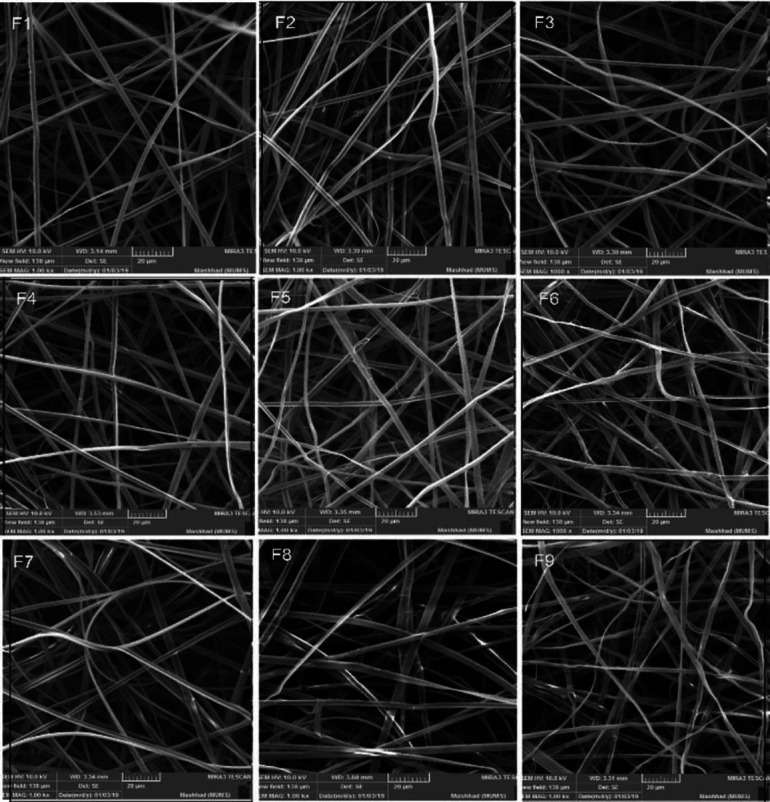
SEM images for the nine formulations in Table 2 with a magnification of 1000 (scale bars 20 μm)

**Figure 2:**
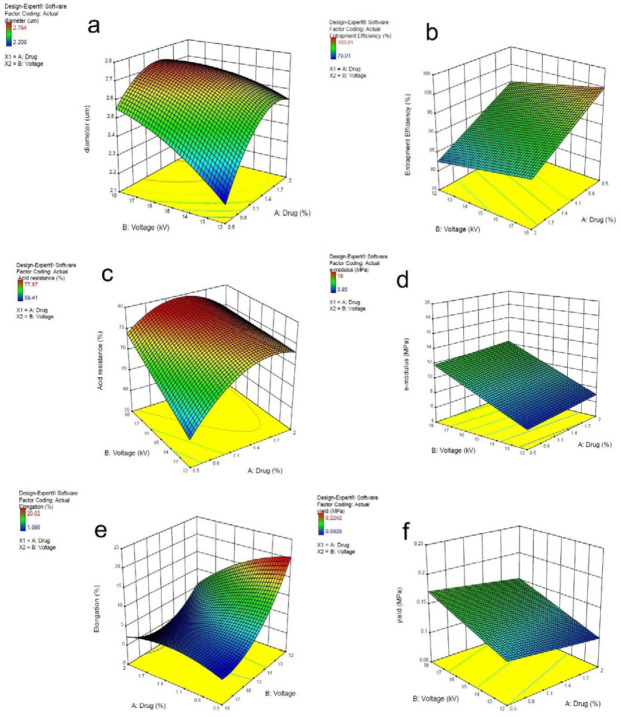
Three-dimensional chart of (a) effect of drug percentage and voltage on the microfibers’ diameter, (b) voltage and drug percentage effect on the percentage of drug content relative to the theoretical value, (c) voltage and drug percentage effect on the percentage of drug residue in the acidic environment, (d) drug percentage and voltage effect on Young’s modulus, (e) drug percentage and voltage effect on elongation, and (f) drug percentage and voltage effect on yield stress

The SEM images of the constructed microfibers are shown in Figure 1, and the mean diameter of microfibers is shown in Table 3. Microfibers were assessed in terms of homogeneity and diameter. To investigate the effects of drug percentage and voltage on microfibers diameter, the Design-Expert software was used. The results are shown in the three-dimensional Figure 2a. The determination coefficient was equivalent to 0.952, and the response surface diagram was obtained by the mathematical equation (2):

Diameter=-0.334176+(1.27026×Drug)+(0.248806× Voltage)+(-0.032333×Drug×Voltage)+(-0.259259×Drug^2^)+ (-0.005759×Voltage^2^)

(2)

***Entrapment efficiency determination***

The drug content in the samples was determined, and the results are available in Table 3. For studying the effect of different variables of drug percentage and voltage on the percentage of drug content concerning the theoretical content, the Expert-Design software was used. The results are shown in the three-dimensional Figure 2b. The determination coefficient was evaluated and was equivalent to 0.694, and the response-surface diagram was determined through the mathematical equation (3):

Entrapment efficiency (%) = 89.9428 + (-8.3066 × Drug) + (0.7927×Voltage)

(3)

***Resistance to the acidic environment***

After exposure of folic acid in microfibers to an acidic environment, its residue was calculated according to the drug content test. The results are available in the form of the percentage of drug residue in the acidic environment. To investigate the variables that affect drug percentage and voltage on folic acid residue, we used the Design-Expert software package. The three-dimensional diagram is shown in Figure 2c. The coefficient determination was equivalent to 0.767, and the response-surface diagram has been obtained by the following mathematical equation (4):

Residuary (%) = 13.615 + (46.3756 × Drug) + (9111×Voltage) + (-1.27778×Drug×Voltage) + (-9.24444×Drug^2^) + (-0.00833×Voltage^2^)

(4)

***Tensile strength test***

The mechanical strength of electrospun samples was calculated through the force-displacement diagram. Table 4 shows the mean of Young’s modulus, yield stress, and elongation for each sample. To investigate the effect of drug percentage and voltage on Young’s modulus, yield stress, and elongation, the Design-Expert software package was used. Their results are shown in the form of three-dimensional diagrams in Figure 2d-f.

The determination coefficient for Figure 2e is equal to 0.856, and the response-surface diagram has been obtained by the following mathematical equation (5):

Yield stress = 187.717 + (-20.2285 × Drug) + (-20.4083 × Voltage) + (1.72778 × Drug × Voltage) + (-4.05393 × Drug^2^) + (0.545296×Voltage^2^)

(5)

***Selection of the optimum microfiber***

In this study, drug percentage and voltage were determined as main independent variables, and other parameters were constant during the study. For investigation of the effect of different parameters on the responses and also to determine the optimum formulation, the Design-Expert software package was used. The results of ANOVA for each mentioned response have been presented in Table 5. Optimization was carried out in terms of the minimum average diameter, maximum resistance to the acidic environment, and medium mechanical strength. Formulation F8 was selected as optimal microfiber, accordingly. 

***Drug release from the optimum microfiber***

**Figure 3:**
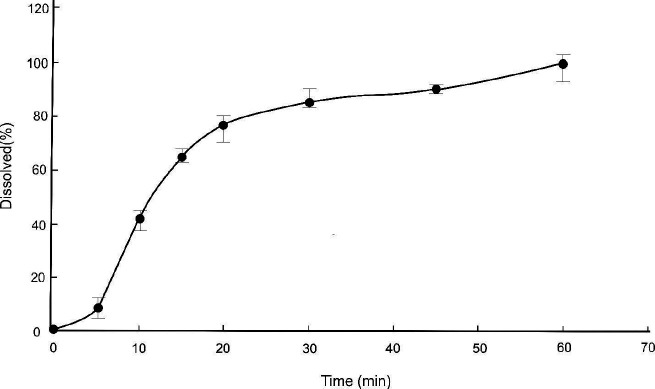
Folic acid release from the microfibers containing 1.25% drug in the voltage of 18 kV at pH = 6.8 (n=3)

According to the results of the drug content test, the release percentage was calculated at different time points. The drug release profile is shown in Figure 3. More than 60% of the drug content has been released in the first 15 min. 

***Differential scanning calorimetry***

Thermal behavior of Eudragit^®^ S 100 powder, folic acid, their physical mixture, and the selected optimum microfiber, and their related DSC thermograms were studied (Figure 5).

***Fourier transform infrared spectroscopy***

FTIR analysis was conducted to detect possible interactions of folic acid and Eudragit^®^ S 100 in the microfibers. The FTIR diagrams of folic acid powder, Eudragit^®^ S 100 powder, Eudragit^®^ S 100 and folic acid physical mixture, and Eudragit^®^ S 100 microfiber containing folic acid are shown in Figure 6, and the prominent peaks are also demonstrated. 

***X-ray diffraction analysis***

The XRD from the 2θ view was obtained for folic acid powder, Eudragit® S 100 powder, Eudragit^®^ S 100 and folic acid physical mixture, and Eudragit^®^ S 100 microfiber containing folic acid. The folic acid diagram shows sharp and distinct peaks, unlike the three other diagrams (Figure 7). 

**Table 1:** Applied factors and levels in experimental design with the box-Behnken design

Levels			Factors
+1	0	-1	
0.5	1.25	2	Folic acid (%)
12	15	18	Voltage (kV)

**Table 2:** Formulations and different conditions of spinning solutions according to experimental design

Voltage (kV)	Folic acid to polymer ratio **(%)**	Formulations
12	0.5	F1
12	1.25	F2
12	2	F3
15	0.5	F4
15	1.25	F5
15	2	F6
18	0.5	F7
18	1.25	F8
18	2	F9

**Table 3:** Measured diameter of microfibers from SEM images, drug content relative to the theoretical value, and drug residue in the acidic environment (n=3

**Drug residue in the acidic environment** **(Mean ± SD)**	**Entrapment efficiency (%)** **(Mean ± SD)**	**Microfiber’s diameter (µm)** **(Mean ± SD)**	**Drug% in microfibers and voltage**	**Formulation**
59.41± 3.73	96.11± 5.19	2.209±0.592	0.5% 12	F1
77.87± 6.21	86.28± 4.76	2.538±0.499	1.25% 12	F2
70.32± 3.8	89.3± 3.81	2.521±0.609	2% 12	F3
67.91± 5.24	95.77± 4.06	2.415±0.640	0.5% 15	F4
74.18± 4.27	90.63± 4.29	2.630±0.704	1.25% 15	F5
76.03± 2.86	79.01± 7.78	2.699±0.626	2% 15	F6
76.23± 5.48	103.91± 4.03	2.562±0.615	0.5% 18	F7
76.32± 0.29	91.95± 6.04	2.764±0.834	1.25% 18	F8
75.64± 2.10	90.10± 3.60	2.583±0.688	2% 18	F9

**Table 4:** Results of the mechanical tests of different fibers (n=3)

**Formulation**	**Drug% in microfibers and voltage**	**Sample thickness** **(um)** **(Mean ± SD)**	**Young’s modulus (MPa)** **(Mean ± SD)**	**Yield stress** **(MPa)** **(Mean ± SD)**	**Elongation (%)** **Mean ± SD)** **)**
F1	0.5% 12	0.244±0.006	7.19±1.320	0.1351±0.0031	20.02±1.53
F2	1.25% 12	0.304±0.011	6.83±0.362	0.1060±0.0131	19.7±2.71
F3	2% 12	0.227±0.011	7.04±1.262	0.0928±0.0108	2.63±0.76
F4	0.5% 15	0.366±0.055	5.85±0.914	0.1001±0.0050	4±0.28
F5	1.25% 15	0.313±0.011	11.07±1.874	0.1636±0.0111	3.4±0.48
F6	2% 15	0.450±0.027	10.45±0.858	0.1429±0.0161	3.22±0.25
F7	0.5% 18	0.209±0.016	10.45±2.971	0.1637±0.0114	4.14±1.17
F8	1.25% 18	0.303±0.018	19±3.66	0.2242±0.0353	1.896±0.14
F9	2% 18	0.483±0.020	7.73±0.789	0.1056±0.0029	2.3±0.16

**Table 5:** ANOVA results of studied responses for different fiber formulations

	**Source**	**Sum of square **	**DOF**	**Mean square**	**F-value**	***P*** **-Value**	**Significance**
Diameter		0.6331	8	0.07914	0.1867	0.9897	ns
	Residual (within formulations)	7.629	18	0.4238			
	Total	8.262	26				
Entrapment efficiency		1148	8	143.5	5.94	0.0008	***
	Residual (within formulations)	434.8	18	24.16			
	Total	1583	26				
Acid resistance		846.8	8	105.9	6.253	0.0006	***
	Residual (within formulations)	304.7	18	16.93			
	Total	1152	26				
Young’s modulus		388.5	8	48.56	13.92	P<0.0001	****
	Residual (within formulations)	62.79	18	3.488			
	Total	451.3	26				
Elongation		1326	8	165.8	123.9	P<0.0001	****
	Residual (within formulations)	24.10	18	1.339			
	Total	1351	26				
Yield stress		0.04297	8	0.005372	23.14	P<0.0001	****
	Residual (within formulations)	0.004179	18	0.0002322			
	Total	0.04715	26				

Significant at the 5% level (*P<*0.05)

**Figure 4:**
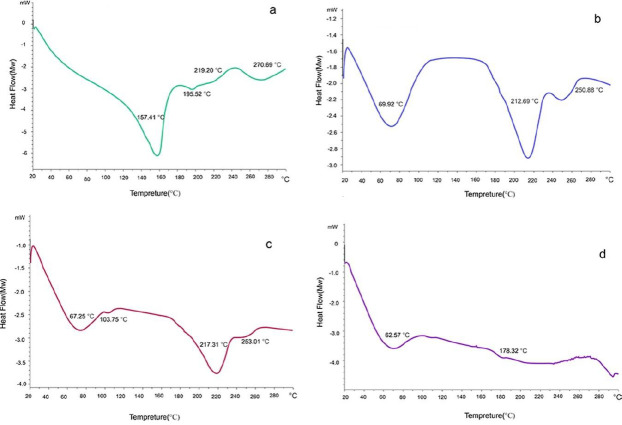
Differential scanning calorimetry thermograms of folic acid powder (a), Eudragit^®^ S 100 Powder (b), Eudragit^®^ S 100 and folic acid physical mixture (c), and Eudragit^®^ S 100 microfiber containing folic acid (d)

**Figure 5:**
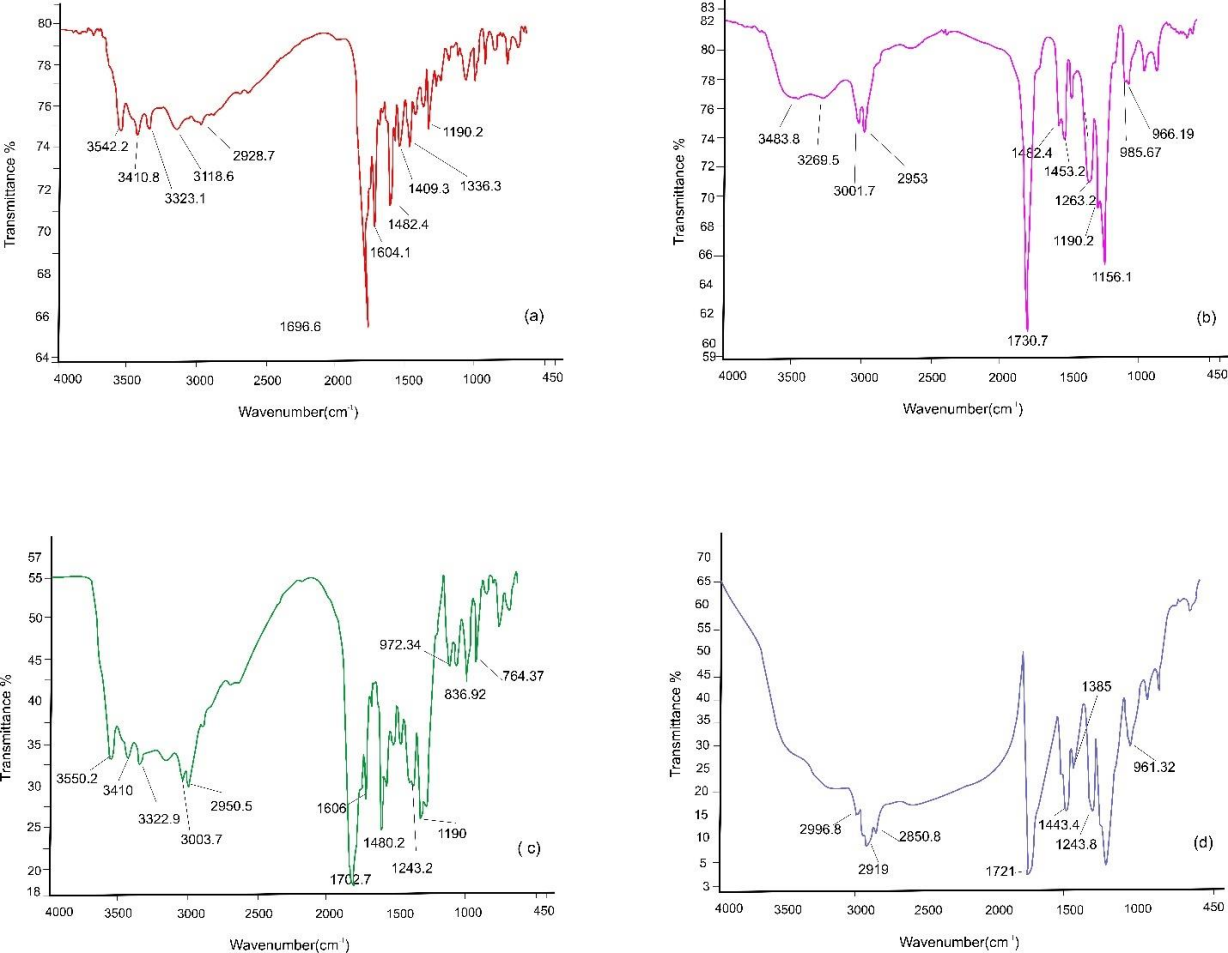
Fourier transform infrared spectrograms of folic acid Powder (a), Eudragit^®^ S 100 Powder (b), Eudragit^®^ S 100 and folic acid physical mixture (c), and Eudragit^®^ S 100 Microfiber containing folic acid (d)

**Figure 6:**
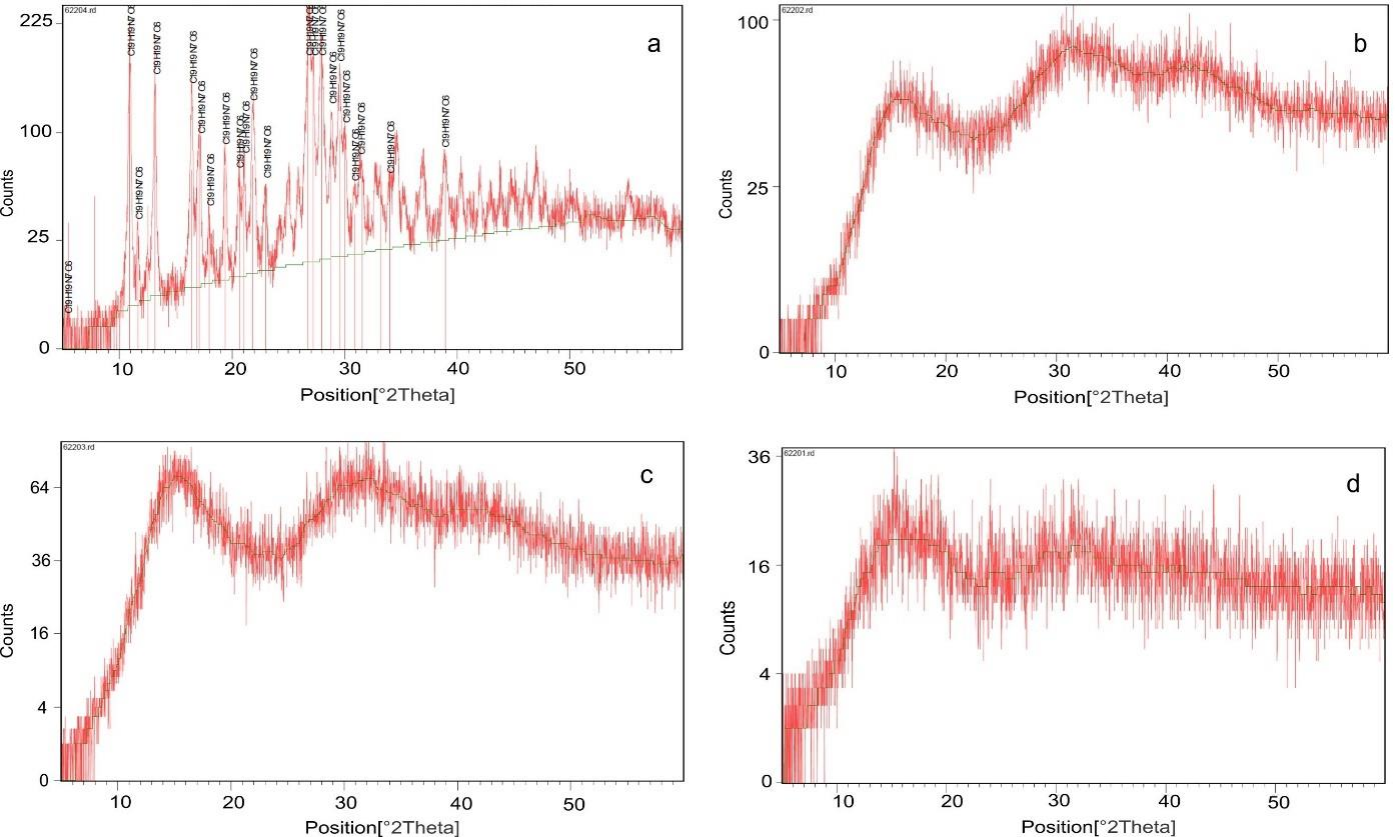
X-ray diffraction diagram of folic acid powder (a), Eudragit^®^ S 100 powder (b), Eudragit^®^ S 100 and folic acid physical mixture (c), and Eudragit^®^ S 100 Microfiber containing folic acid (d)

## Discussion

SEM micrographs of the electrospun microfibers indicated a uniform and bead-free structure without any drug crystals, which validate the entrapment of drugs in the microfibers. The mean diameter of microfibers was not in the nanometer range due to the incomplete dissolution of folic acid in the polymeric solution. The results showed that in the constant concentration of the drug, as voltage elevates, the microfibers’ diameter increases but this effect was not statistically significant. Other studies also validated the direct impact of the applied voltage on the nanofibers’ diameter (31,32). It could be rational because as the voltage increases, the volume of the droplet at the needle tip decreases, so the Taylor cone will recede, which increases the amount of ejected fluid and increases the flow of polymer solution leading to a larger diameter of the microfibers. By increasing the drug concentration, microfibers’ diameter increases proportionally. It may be attributed to increased apparent viscosity or conductivity of the polymeric solution upon increasing drug concentration (33)micron- and nanofibers can be obtained from polymer solutions under a very high electrical field. A special challenge is to produce bead-free uniform fibers since any minor changes in the electrospinning parameters such as slight variations in the polymer solutions and/or electrospinning experimental parameters may result in significant variations in the final nanofiber morphology. Furthermore, it is often not trivial at all to obtain reproducible uniform electrospun nanofibers for the optimized electrospinning conditions. Here we report that the conductivity of the solvent is the key factor for the reproducible electrospinning of uniform polystyrene PS. 

According to Table 3, the entrapment efficiency was above 79%. Drug percentage and voltage significantly affect entrapment efficiency. Drug percentage to voltage ratio has more effect on entrapment efficiency. According to Figure 2(b), by increment of drug amount in a constant voltage, the entrapment efficiency decreased. The highest entrapment efficiency belongs to the samples with 0.5% drug, which decreased by the increment of drug percentage. The result could be attributed to the fact that Eudragit^®^ S 100 microfibers can protect a limited amount of drugs. In high levels of the drug, the remaining drug might be placed on the surface of polymeric microfibers, and due to folic acid susceptibility to oxidation and light, the exposed drug would be destroyed. (34). 

The analysis of resistance to the acidic environment demonstrated that the produced microfibers have acceptable resistance to the acidic environment, as the percentage of drug residue in the acidic environment was between 59.41 and 77.87. Drug percentage and voltage can significantly affect the acidic resistance. As it has been shown, in constant drug percentage, by the voltage increment, resistance to acidic environment increases. Moreover, in constant voltage, the higher drug percentage leads to higher acidic resistance.

The fiber diameter is an essential factor in the rate and amount of drug released (35). According to Figure 2(a), by elevation of voltage and drug percentage, the microfibers’ diameter increases, which leads to a decrease in the surface to volume ratio, and less drug will be exposed on the surface of microfibers. As a result, less amount of drug will be exposed to an acidic environment, which may cause more resistance to the acidic environment. 

Several factors affect the strength of the microfibers, such as diameter, surface morphology, length, orientation, and the cohesion frictional forces between the nanofibers (36). Polymeric films should not be too flexible because too much elongation during cutting and packaging may cause heterogeneity of the film resulting in non-uniformity of drug amount in the nanofilm (37)”ISSN”:”19994923”,”abstract”:”The production of orodispersible films (ODFs. 

Mechanical properties of polymeric films can be defined in terms of Young’s modulus, percentage of elongation, tear-resistance, and tensile strength (38, 39). Various factors like the film-forming factor, type of manufacturing process, film thickness, and type and amount of drug in film formation should precisely be considered for controlling the mechanical strength of the film (40). The result of mechanical tests showed that the variables in this study have a significant effect on elongation, Young’s modulus, and yield stress of microfibers. According to Figures 2(d), 2(e), and 2(f), by voltage amplification, elongation decreased, but Young’s modulus and yield stress elevated. Drug percentage did not have any effect on these mentioned factors. In other words, by voltage amplification, microfiber resistance increases against deformation and elasticity decreases. By voltage amplification, microfiber diameter increases, which leads to more resistance of microfiber against deformation. It has been shown that by reduction of microfiber diameter, tensile strength would decrease, and by increasing the microfibers’ diameter, the fibers will be more resistant against deformation (41). In another study, it was confirmed that by polymer ratio increment, the diameter of electrospun nanofibers increased, so Young’s modulus and film strength increased (42). After optimization of the formulations in terms of the maximum entrapment efficiency, the maximum resistance to the acidic environment, strength, and mean elasticity of film, the F8 formulation was selected, as optimum microfiber contained 1.25% of folic acid which was produced in the voltage of 18 kV. The optimum formulation (F8), demonstrated in Figure 1, has a bead-free structure. The entrapment efficiency of the formulation was 91.95±6.04% which was reasonable. The drug residue in an acidic environment was 76.32±0.29 which confirms the microfibers’ capability in the protection of the drug from the acidic environment of the gastrointestinal tract.

In the release test (Figure 4), as mentioned, about 60% of the drug was released in the first 15 min, followed by a slower release (in 60 min). For the homogeneous microfibers, the drug release rate decreases upon time, as the drug has to traverse a longer distance to diffuse from the microfiber (43). In the early phases of the dissolution test, the release rate was higher due to drug diffusion from the microfiber surface, and the rate of drug release reduced gradually over time (44-46). 

According to the DSC diagrams, Eudragit^®^ S 100 has three peaks at 69.92 ^°^C (a broad endothermic peak), 212.69 ^°^C, and 250.88 ^°^C. The peak at 69.92 ^°^C is related to the melting point of the polymer which has been also confirmed by some other studies (47, 48). The 212.69 ^°^C and 250.88 ^°^C peaks are related to the structural destruction of the polymer. In the folic acid diagram, a broad endothermic peak at 157.41 ^°^C indicates the melting point of this drug, and three other peaks at 195.52 ^°^C, 219.20 ^°^C, and 270.69 ^°^C are also observed. The endothermic peak at 195.52 ^°^C validates the loss of amide and acid functionalities and 219.2 ^°^C and 270.62 ^°^C endothermic peaks, confirm the deterioration of folic acid and its conversion from the crystalline form to the amorphous form. Some studies confirm that folic acid structural destruction is above 200 ^°^C (49). According to the diagram of the physical mixture of the polymer and drug, the two endothermic peaks at 67.25 ^°^C and 217.31^°^C are related to the melting point of the polymer and the destruction of the polymer, respectively. Elimination of folic acid peak in this diagram could be attributed to conversion of folic acid crystals to the amorphous form. In the thermogram of the produced microfiber, an endothermic peak is demonstrated at 62.57 ^°^C, which could be attributed to the amorphous form of the drug in the electrospun microfibers.

In the folic acid FTIR diagram in Figure 6, a peak is demonstrated at 3542.2 ^°^C, which is related to the stretching vibration of O-H in the glutamic acid section, and two peaks at 3410.8 ^°^C and 3323.1 ^°^C, which are related to the stretching vibration of N-H at pteridine ring in folic acid structure. The peak at 1696.6 ^°^C is attributed to the stretching vibration of the C=O group of carboxylic acid. The peak at 1604.1^°^C is related to the bending of the N-H group and the peak at 1482.4 ^°^C is relevant to the phenyl ring. In the range of 2000–2600 ^°^C, no peaks were found (50). The peak at 1409.3 ^°^C is related to the deformation of the O-H bond in the phenyl skeleton (51) and the peak at 2928.7 ^°^C is attributed to the C-H bond of the folic acid structure (52).

The interpretation of the IR spectrum of Eudragit^®^ S 100 is complicated as this polymer is a mixture of polymers with different molecular weights. The peak at 3483.8 ^°^C is related to the tension in the hydrogen bond of the hydroxyl group. The peak at 1730.7 ^°^C is relevant to the esteric carboxylic group of C=O (44). The peak at 2953 ^°^C is related to the carboxylic acid group of O-H, and the peak at 1453.2 stands for –CH3 (45). The peak at 3269.5 ^°^C is related to the O-H bond (53). The 1263.2, 1190.2, and 1156.1 ^°^C peaks are related to the tension of C–(C=O)–O and O–C–C in Eudragit^®^ S 100 structure (54). In the IR spectrum of the physical mixture of polymer and drug, both peaks of polymer and drug are demonstrated concurrently. The peaks at 3410, 3322.9, and 1606 ^°^C are related to folic acid. The 3003.7, 2950.5, and 1243.2 ^°^C peaks are related to the polymer. The two peaks of 1480.2 and 1190 ^°^C are related to folic acid and polymer. The peak at 1702.7 ^°^C is the result of interference between the two prominent peaks of drug and polymer. In the FTIR spectrum of the produced fiber, alterations in peaks are most probably related to OH stretching modes of the hydroxide basal layer or water molecules exhibiting a broad absorption peak, showing lack of interference between the drug and polymer in the microfiber (55).

The sharp and distinct peaks in Figure 7(a), are related to the XRD spectrum of folic acid, which is representative of the crystalline form of the drug (56). According to this study, the XRD spectrum of Eudragit^®^ S 100 has not shown any sharp and distinct peaks, which is representative of an amorphous structure (57). Both physical mixtures of drug and polymer and microfiber spectrum also showed that folic acid has lost its crystallinity and converted to its amorphous form, both of which confirm the DSC results.

## Conclusion

In this study, electrospun microfibers of Eudragit^®^ S 100 containing folic acid were produced by the electrospinning method, and their properties were tested. According to the obtained results, the optimum formulation was homogeneous, bead-free fiber with a drug to polymer ratio of 1.25%, and was produced by the voltage of 18 kV. The entrapment efficiency for this formulation was above 90% with an acidic resistance of higher than 70%. The release pattern evaluation of the optimum formulation demonstrated a controlled and acceptable release of the drug in the phosphate buffer (pH 6.8) which could be a promising result for folic acid to bypass gastric acidic environment. The DSC and XRD evaluations confirmed the amorphous form of folic acid in the fiber structure validating the higher dissolutions rate of this drug. The FTIR test shows no chemical bond between folic acid and Eudragit^®^ S 100. Overall results indicate the selected formulation as a potential candidate for further stability and bioavailability studies.

## Authors’ Contributions

MRA designed and supervised the experiments; AHF, AA, MRA collected and processed the data and performed the experiments; AA, MRA discussed the results; PI, NR prepared the manuscript draft; AA, MRA, NR, PI revised and edited the article; PI, NR, AHF, AA, MRA Final approved of the version to be published.on to be Published; MRA Supervision, Funding Acquisition.

## Conflicts of Interest

The authors declare that there are no conflicts of interest.
